# Febrile Seizure: Demographic Features and Causative Factors

**Published:** 2012

**Authors:** Hamed ESMAILI GOURABI, Elham BIDABADI, Fatemeh CHERAGHALIPOUR, Yasaman AARABI, Fatemeh SALAMAT

**Affiliations:** 1 Medical Student, Guilan University of Medical Sciences, Rasht, Iran; 2 Assistant Professor of Pediatric Neurology, Guilan University of Medical Sciences, Rasht, Iran; 3 Epidemiologist, Research vice-chancellorship, Guilan University of Medical Sciences, Rasht, Iran

**Keywords:** Febrile seizure, Children, Respiratory tract infections

## Abstract

**Objective:**

Because of geographical and periodical variation, we prompted to determine the demographic features and causative factors for febrile seizure in Rasht.

**Materials & Methods:**

In this cross-sectional study, all 6–month- to 6-year-old children with the diagnosis of febrile seizure admitted to 17 Shahrivar hospital in Rasht, from August, 2009 to August, 2010 were studied. Age, sex, family history of the disease, seizure types, body temperature upon admission and infectious causes of the fever were recorded. All statistical analysis was performed with SPSS software, version 16.

**Results:**

Of the 214 children (mean age, 25.24±15.40 months), 124 were boys and 109 had a positive family history. Complex seizures were seen in 39 cases. In patients with a complex febrile seizure, 59% had the repetitive type, 20.5% had the focal type and 20.5% had more than 15 minutes duration of seizures. Most of the repetitive seizures (78.3%) occurred in patients under 2 years old; the difference between under and over 2-year-old patients was statistically significant. Study results did not show significant differences between the two genders for simple or complex seizures. The mean body temperature upon admission was 38.2±1.32◦C (38.31±0.82 degrees in boys and 38.04±1.78 in girls). Upper respiratory infections were seen in most patients (74.29%). All cases of lower respiratory infections were boys. There was a statistically significant difference between boys and girls in causes of fever.

**Conclusion:**

Most of the children had a positive family history and the most common causative factor was upper respiratory infection.

## Introduction

Febrile seizure is the most common type of convulsive disorder and one of the most prevalent cause of emergency hospital admission in children (1) Several studies have attempted to detect risk factors associated with seizure (2). Febrile seizure stems from genetic and environmental factors (3). Although the main cause for this type of seizure is not yet recognized, the most important identified risk factor for febrile seizure is the presence of a positive family history in immediate family members (4). In 2008, a study demonstrated that 25.7% of these patients had a positive family history (5). In addition, perinatal events and developmental delay are the other factors (6). Male gender, breast- feeding duration, high body temperature, low birth weight, high blood bilirubin or neonates whose mothers consume alcohol and smoke cigarettes (7) are related to febrile seizure. Bacterial and viral infections are considered as significant factors (4). Viral diseases are some of the important causes for febrile diseases present in the form of diarrhea, vomiting, acute upper respiratory tract infection (URI), acute otitis media (AOM) and urinary and digestive infections (8). Febrile seizures due to infection were predominantly upper respiratory tract infection in which the cause was unknown or bacterial in the first half of the twentieth century (9). Viral infections were rarely recognized. More recent studies attributed the majority of pharyngeal infections to viruses. Some recent studies showed that the most frequent causative infection in other countries is otitis media, as Hoseini Nasab et al. showed in Kerman and Ashrafzade et al. in Mashhad (8, 10), but otitis media is much less seen in our practice with children suffering from febrile seizure. Because of the conflicting results of previous studies about causative factors of febrile seizure and because of geographical, periodical and even seasonal variation, we prompted to determine the demographic features and causative factors for seizure in Rasht.

## Materials & Methods

In this cross-sectional study, the medical data of all 6-month- to 6–year-old children with the diagnosis of febrile seizure admitted to clinics or hospitalized in 17 Shahrivar hospital in Rasht, from August 2009 to August 2010, were studied.

Patient’s data including age, sex, family history of disease, seizure types (simple or complex) and complex seizure subtypes such as focal, repetitive, or a duration of more than 15 minutes, body temperature upon admission, and infectious causes of fever were recorded in specific self-formatted questionnaires.

All children with a seizure due to central nervous system infections, a past medical history of seizure, and co-orbidity with chronic diseases or electrolyte imbalance were excluded from our study. We obtained written informed consent from all parents for inclusion in the study. The Ethics Committee of Guilan University of Medical Sciences approved the study.

Normality of data distribution was assessed by the Kolmogorov-Smirnov test. Discrete variables are expressed as counts (%) and compared using Chi-square tests. Continuous variables are expressed as mean ± SD and compared by means of the unpaired, two-sided t test. Adjusted odds ratios and 95% Wald confidence intervals were calculated based on these models.

Statistical significance was set at P<0.05. SPSS 16 for Windows (SPSS Inc., Chicago, Illinois, USA) was used for statistical analysis.

## Results

We studied a total of 214 children with febrile seizure, of which 124 cases (57.9%) were male and 90 (42.1%) were female. The mean age of the patients was 25.24±15.40 months, and 128 children (59.8%) were under 2 years. The mean age for male and female were 25.62±15 and 25.13±16.11 months, respectively (P= 0.81). One hundred and nine children (50.9%) had a positive family history. 

Simple febrile seizures were seen in 175 patients (81.8%) and complex seizures in 39 cases (18.2%). Of the patients with complex febrile seizure, 23 cases (59%) had the repetitive type, eight cases (20.5%) had the focal type, and eight cases had more than 15 minutes duration of febrile seizures. Most of the complex seizure cases with repetitive type (78.3%) occurred in patients under 2 years old. In this aspect, the difference between the two age groups (under and over 2 years) was statistically significant (P=0.02). Study results did not show significant difference between two genders for simple or complex seizures.

The mean body temperature upon admission was 38.2±1.32 degrees centigrade that was 38.31±0.82 degrees centigrade in boys and 38.04±1.78 degrees centigrade in girls (P =0.15). 

Data on patient’s underlying causes for fever are provided in Table 1. Upper respiratory tract infections were seen in most patients (159 cases, 74.29%). Underlying causes in 78.22% of the boys were upper respiratory tract infection, while it was seen in 65.5% of the girls. Lower respiratory tract infection was not seen in any of the girls, but three boys (2.4%) experienced it, so all cases of lower respiratory tract infections were boys. The second most common cause of fever was gastroenteritis in the boys and undiagnosed cases in the girls. Totally, there was a statistically significant difference between the boys and girls regarding the cause of fever (P=0.016).

**Table 1:** Underlying Causes of Fever

	**Male (n=124)**	**Female (n=90)**	**Total (n=214)**	**P-Value**
**Upper Respiratory Tract Infection**	97 (78.22%)	59 (65.5%)	156 (72.89%)	0.016
**Gastroenteritis**	13 (10.5%)	12 (13.3%)	25 (11.68%)	
**Lower Respiratory Tract Infection**	3 (2.4%)	0	3 (1.40%)	
**Other causes (Urinary Tract Infection, Roseola, Acute Otitis Media, Appendicitis, Chicken Pox)**	5 (4.03%)	3 (3.3%)	8 (%3.73)	
**Undiagnosed Cases**	6 (4.8%)	16 (17.8%)		

## Discussion

Febrile seizure is the most common seizure in childhood (11). Occurring in 2-7% of the children aged 6 months to 6 years (12). In this study, the mean age of the patients was 25.24±15.4 months, but in some studies, 23.68 months was the approximate estimated age (13-14). Hassanpour estimated that 35.2% of the children experienced their first febrile seizure in the age of 1 year (15). Fallah mentioned 66% of the children with this type of seizure were smaller than 2 years and 6% of them were older than 4 years old (13). In our study, 59.8% of the children were smaller than 2 years and 78.3% of them had complex febrile seizure. We have observed a significant difference in children with repetitive complex febrile seizure between the two age groups, under and above 2 years old, as most of them were younger than 2 years (P=0.003).

In this study, 124 cases with febrile seizure were boys (57.9%) and the remainder were girls (42.1%). A definite male predominance was detected for febrile seizure in our study (p=0.21). These results are similar to the findings of a study conducted by Habib et al. in Pakistan; he found that the male gender had a 1.3 times greater risk of febrile seizure (16). This is also supported by a study performed by Abaskhanian et al. in 2010, that quoted a slight predominance of febrile seizure in males (17).

Mahyar et al. in 2010 found that gender is an important factor in febrile seizure; in his study, 66% of the infants with febrile seizure were boys (18). Ashrafzade et al. also noted that febrile seizure is more frequent in boys than girls (8). 

In this survey, 81.2% of the patients had simple and 18.2% had the complex form of febrile seizure. This was also stated by Hosseini Nasab et al. In his study on 460 infants with febrile seizure, simple and complex form of febrile seizure were 76/4% and 23/6%, respectively (10).

n our study, 50.9% 0f affected children had positive family history of febrile seizure. In other Iranian studies, performed in Boushehr (59%), Kashan (55%) and Kerman (50%), positive family history was identified as most remarkable risk factor in these children (10, 20-21), but it was estimated about 14 % in another study (8). 

Viral disease manifested as upper respiratory tract infection (URI) and gastroenteritis, is one of the causes of febrile disease. Khodapanahande in his survey in Tehran found that nonspecific viral disease is the main cause of febrile seizure, with a frequency of 82% (14). Mahyar et al. in 2010 and Kolahi et al. in 2009 encountered respiratory tract infection as the most prevalent cause of febrile seizure (18-19). In the present study, upper respiratory tract infection was the main reason of febrile seizure in 74.29% of the cases and gastroenteritis (11.68%) was the second cause of fever. URI was the most important cause of fever in other studies (6, 10, 13, 17, 22). In some studies, gastroenteritis was ranked second grade after URI (6, 13). Hassanpour found that URI is the first most common cause of febrile seizure and lower respiratory tract infection is (LRI) the second most common cause (15). In a study carried out by Salehi Omran et al. in 2008, the etiology of fever was identified in 85.3% of the cases and the etiology remained unknown in 14.7% of the cases. In this study gastroenteritis was identified in 27.3% of the cases (5). In another study in Mashhad, 36% of the patients with febrile seizure had AOM, and URI and GI were the next frequent (8); this result is in contrary to our study, in which AOM was not a significant cause of fever (1.4%). Hosseini Nasab in a study performed in Kerman indicated that AOM with a 40 percent prevalence is the important inducing factor of fever (10). 

**In conclusion, **in this study, the occurrence of febrile seizures was higher in boys than girls. In addition, most of the children had a positive family history of febrile seizure, and the most common causative factor was the upper respiratory tract infection. There was a statistically significant difference between boys and girls regarding the cause of fever. Repetitive type of complex seizure was seen mostly in patients less than 2 years; the difference between patients under and over 2 years was significant.
